# The diffusion of sustainability and *Dingle Peninsula 2030*

**DOI:** 10.14324/111.444/ucloe.000052

**Published:** 2022-11-08

**Authors:** Evan Boyle, Connor McGookin, Deirdre de Bhailís, Brian Ó Gallachóir, Gerard Mullally

**Affiliations:** 1 MaREI, The SFI Research Centre for Energy, Climate and Marine, Cork, Ireland; 2 Department of Sociology and Criminology, University College Cork, Cork, Ireland; 3 School of Engineering, University College Cork, Cork, Ireland; 4 Dingle Hub, Dingle Peninsula, Co. Kerry, Ireland

**Keywords:** engaged research, collaboration, sustainability, socio-technical transitions, climate action, diffusion, community, *Dingle Peninsula 2030*, climate

## Abstract

Instilling a collaborative approach can widen participation to a range of stakeholders, enabling the diffusion of sustainability and increasing local capacity to meet decarbonisation targets to mitigate against climate change. *Dingle Peninsula 2030* has emerged as an international case study of a collaborative regional sustainability project, whereby a wide range of initiatives, beyond the initial remit of the project, have emerged in the area. This holistic scale of action is required for effective climate action. Using the Sustainable Development Goals (SDGs) as a framing, the interrelated nature of climate action has been shown through this study. In setting out to undergo energy projects a wide range of new initiatives emerged as community members became engaged in the process. Initiatives have emerged related to energy, transport, agriculture, education, tourism and employment, in what we have coined the ‘diffusion of sustainability’.

## Introduction

In outlining the case study of *Dingle Peninsula 2030*, this paper highlights how community initiatives can offer an insight into the future potential of such projects to elicit an acceleration of the transition to a low-carbon society. In Ireland, community-led initiatives have gained less attention than their European counterparts [1]. The emergence of a wide array of community-led initiatives throughout Europe was referenced almost a decade ago [2]. In Ireland, however, despite being the second-largest per capita carbon dioxide (CO_2_) polluter in Europe [3], we have witnessed only a few isolated examples. Of the few Irish studies, it is suggested that such projects can engender high levels of citizen buy-in and can create social contexts conducive to involvement [4]. Local needs such as job creation and economic reasoning can act as leverage points for local action, rather than global environmental concerns [2]. People are recruited to such initiatives by others in the community, and continued involvement occurs due to community gains rather than individual gains [5].

Community led initiatives can play an important role in bringing about the emergence of more widespread action for decarbonisation in responding to climate change, with regards to both mitigation and adaptation [6]. The need for the mass mobilisation of community-led initiatives represents the potential to usurp ‘dominant individualist and consumerist lifestyles’ which ‘run counter to community collectivism’ [7]. Ireland has a long history of area-based community or co-operative partnerships [8,9]. In a rural context, the tradition of ‘Meitheal’ – an old Irish term which denotes collective local structures for working together in rural contexts – exists. Community contexts for co-operation and collaboration offer potentially fertile grounds for action in the face of national and international inertia on decarbonisation and sustainability more broadly.

The case study under investigation is *Dingle Peninsula 2030* or *Corca Dhuibhne 2030* (Irish translation), a collaborative project which is seeking to transition a region in the peripheral south-west of Ireland to a low-carbon, climate resilient, sustainable community by 2030. A transdisciplinary, engaged research approach has been taken by the research team in working with the *Dingle Peninsula 2030* collaborative committee. This action-orientated approach seeks to support local sustainability initiatives while drawing out valuable learnings to inform the wider transformation needed [10]. The key research question emerging is: what has been the contribution of the project to sustainability goals? This is important to evaluate the contribution of the research approach [11], and also track progress toward vital sustainability goals such as reducing emissions [12].

It is widely acknowledged in the literature that more action-orientated research is needed to support sustainability goals [10,13,14]. However, there have been very limited examples of how to monitor or measure the contribution of such research projects and broader collaborative efforts to said goals. Understanding the impact of the project poses a serious methodological challenge due to its emergent and evolving nature. Furthermore, the exact contribution of different interventions or actors is hard to classify. Horlings et al. [15] from a review of 15 ‘embedded’ research projects note that there is a need to develop new metrics of impact for such processes.

This paper makes effort to address this issue by using the sustainable development goals (SDGs) to map the projects that have emerged in the case study area. The unique approach leads to the concept of the diffusion of sustainability as a means to understand impact. It points to a wide breadth of novel initiatives beyond the original objectives of the project. As would be expected the core focus of the *Dingle Peninsula 2030* partnership on energy and climate is common in many of the initiatives. However, we have also seen developments in the areas of education (SDG 4), decent work (SDG 8) and innovation (SDG 9), which reflect the broader concerns of the community. This points to the importance of such place-based approaches to align vital sustainability objectives with community goals.

## *Dingle Peninsula 2030* and the diffusion of sustainability

### Case study background

The Dingle Peninsula has a population of 12,500. As outlined by McGookin et al. [12], the Peninsula has a lower proportion of high social class categories of employment than the national average. There are limited manufacturing-based employment opportunities leading to a small number of people in occupations classified as manual and semi-skilled. Relatively speaking there is a higher percentage of ‘own-account workers’ related to the tourism industry, and there is a higher significance of both agriculture and fishing. Both sectors, however, are situated within the unsustainable current practices and historical timelines which have led to the contemporary situation. The *Dingle Peninsula 2030* project emerged as an initiative seeking to facilitate the incubation of decarbonisation initiatives, with the central aim of bringing sustainable employment to the area. It has employed a novel governance structure through the formation of a collaborative committee. This committee consists of representatives from a local not-for-profit interested in enterprise (Dingle Hub) and community development [North East West Kerry Development (NEWKD)], as well as Ireland’s national electricity distribution system operator (ESB Networks) and our research institute (MaREI). This transdisciplinary configuration is grounded within the local community, whilst also having capacity at a national level. From a research perspective the *Dingle Peninsula 2030* initiative has been investigated with relation to social network analysis [16], the engaged research approach deployed, the collaborative governance structure [17] and participatory methods for energy system modelling.

Despite emerging as recently as 2018, *Dingle Peninsula 2030* acts as a community-based, transdisciplinary collaboration that has had impact both at a local community level, and on a wider national and international stage. It offers valuable learnings on the real-world transformation process to guide efforts elsewhere [18]. The project has gathered national media coverage, been designated as a living laboratory by the United Nations (UN) and has sent a delegation to the UN Climate Change Conference (COP26) to discuss the role of community-based initiatives for climate action [19]. The case study has been investigated for the period January 2018 up until June 2021.

### Diffusion of sustainability

Niche social and technical innovations are emerging that differ radically from the prevailing socio-technical system and regime but are able to gain a foothold in particular applications, geographical areas or with the help of targeting policy support. Community contexts offer forth the experimental space in which niche innovations can emerge. They can be technical, social or governance related. The global challenge of climate change is experienced within local, community contexts, and as such, these contexts often bring about novel solutions that operate outside of global multi-lateral deliberations, with the potential to provide insights towards policy implementation. While niche innovations often act within specific and unique community contexts, they can be extrapolated from to provide wider learnings. While community action can assist in informing policy developments, there is a need to develop mechanisms which can represent instances of success. Here, the diffusion of sustainability is offered as a framing through which the emergence of novel innovations can be outlined in real time, using the analysis of a case study context.

Recent work on the diffusion of transformative innovations for sustainability [20] has highlighted several different categorisations for situating diffusion. From this a typology of development mechanisms for transformative innovation has been formatted. A number of these have relevance to the case study context presented in this paper. Growing, whereby an initiative quantitatively grows by attracting more participants and funding, can be facilitated by increased social visibility, professionalisation and communication capacities. Replication, whereby ideas or practices are translated into new contexts, can often occur due to ideas percolating in the media or communication channels. Partnering, whereby initiatives share resources and capacities, takes place when collaboration is mutually beneficial. These three typologies have acted to frame our understanding of the diffusion of sustainability in the context of *Dingle Peninsula 2030.*

As such, the diffusion of sustainability is investigated in this study, through an analysis of a collaborative, transdisciplinary approach to sustainable transitions in the community context. Nineteenth century social theorist Gabriel Tarde illustrated the idea of logical laws, whereby innovations which are aligned with the rational logic of a given society are more likely to spread than those which are not [21]. Kinnunen [21] illustrates the significance of the work of Tarde to the establishment and continuity of diffusion research. To him, diffusion is a descriptive concept; ‘the spread of social or cultural properties from one society or environment to another’ [21, p. 432]. Imitation can be seen as a key element in the process of diffusion. An S-shaped curve includes initial level innovators (who themselves have emerged through imitative processes) followed by early adopters, early majority, late majority and laggards. The latter four distinctions can be said to act within the laws of imitation within the diffusion of practice. The link between Tarde’s work and diffusion research has been central to diffusion research in recent decades [21,22].

Diffusion can be seen to occur through three mechanisms: internal influence, external influence and mixed influence. At a most cursory level, internal influence for the diffusion of innovation is concerned with the spread of new ideas and practices through interpersonal communication between interpersonal contacts [23,24]. External influence is diffusion through external sources not related to interpersonal relationships. The media plays an important role in external influence alongside private sector triggers and policy developments. Finally, mixed influence involves both internal and external influence principles whereby both the wider landscape and interpersonal relationships impact the adoption of innovations.

The last number of years has seen a growth in the emergence of research investigating the diffusion of social innovations related to transformations towards sustainability. The investigation of social innovations with relation to isolated rural locations, similar to the case study context under investigation, has recognised the importance of both local and external actors to processes of change in rural locations, but emphasised the central importance of local knowledge and local activities [25,26]. The regional level is understood within the literature to be an important context for the diffusion of sustainability to other locations [27]. The case study outline provided here can contribute to raising awareness of regional innovations for sustainability, and the diffusion mechanisms at play.

Diffusion, in keeping with the foundational work of Rogers [28], can be viewed as a process through which an innovation can be communicated using certain mediums or channels over time to members of a social group or system. Diffusion is understood as a ‘cascading mechanism that leads to cumulative adoption of behaviours by some individuals even while their social position, or the resources associated with them, changes only trivially or remains unaltered’ [29]. Borrowing from Clough et al. [26], sustainability is defined as:

“a process that helps create a vibrant economy and a high quality of life, while respecting the need to sustain natural resources and protect the environment. It expresses the principle that future generations should live in a world that the present generation has enjoyed but not diminished.” [26, p. 30].

Our definition for the diffusion of sustainability is understood to be aligned with the definitions of both Palloni [29] and Clough et al. [26], whereby initiatives, projects or actions which support the creation of a vibrant economy and high quality of life, whilst respecting natural resources and the environment, are cumulatively developed or adopted. This definition builds upon social innovation approaches to transitions whereby the centre of change under investigation is changing social practice rather than technological approaches to innovation [30]. Social innovation as a field of investigation has been rapidly growing within the academic literature in recent times [31].

The SDGs offer a useful mechanism for capturing social innovations, with nine socially-orientated goals represented within the framework (SDG 1, 2, 3, 4, 5, 8, 10, 11, 12) [31]. Our own use of the ‘diffusion of sustainability’ as a heuristic device for capturing the emergence of novel innovations in sustainability transitions is built upon the SDGs. An SDG framing is used to not only represent social innovations but can look towards technological/infrastructural innovations (SDG 7, 9), environmental/ecological innovations (SDG 6, 13, 14, 15), and institutional/governance innovations (SDG 16, 17). The SDGs have been criticised for being hard to quantify, implement and monitor [32]. Some progress has been made with relation to these challenges [33,34], yet they still remain more progressive in their aspiration than in their impact. By using a case study approach to outline the diffusion of sustainability in a regional context, we offer a foundation from which impact can be charted.

## Methods

### A mixed-method approach to the diffusion of sustainability

Within this piece of research, diffusion has been investigated and highlighted through a qualitative approach combining exploratory interviews, ethnographic attendance of events and meetings, and secondary desk research. Also, once compiled, the diffusions outlined have been cross referenced with the primary project manager for *Dingle Peninsula 2030*. The aim is to outline the different emergent projects and initiatives which have developed in light of *Dingle Peninsula 2030* through a process of diffusion. The list of projects is based on those that the four core partners in *Dingle Peninsula 2030* were involved in between January 2018 to June 2021. In addition, effort has been made to map the various spin-off projects led by key local actors that took part in *Dingle Peninsula 2030* events or initiatives and have since sought to champion sustainability projects of their own.

A key challenge associated with this task is the fact it may take years for transformations to take place. In the long term, a more in-depth data-centric approach to diffusion research would be insightful. The development of retrospective historical analysis of social diffusion is an area in which new mechanisms and methods are being developed, informed by contemporary network concepts and models [35]. Such approaches would provide useful means of investigation if one was to set about interpreting the diffusion of sustainability in Dingle at a later date. Here, however, we have sought to develop an approach which can chart and categorise diffusion in real time, as part of a rapidly evolving project.

### Developing a process for investigating diffusion

A range of different diffusion models has been developed within sociology and applied to a range of different social situations. There is, however, an issue with regards to the suitability of these models in certain situations, due to the need to internalise theories, assumptions and constraints within the models with relation to a range of factors such as socio-economic status, preferences, decision-making processes and individual constraints amongst other factors. As has been noted, ‘the conditions for identification of a diffusion process from observables are quite hard to meet, much harder than what is normally implied in traditional applications of diffusion theories and models to sociological and demographic analysis’ [29].

Studying the diffusion of sustainability, in relation to environmental issues, is a well-developed field within tourism [36–38]. For our purposes, however, an investigation into the diffusion of sustainability in relation to regional transition projects is missing from the literature. It builds upon the three typologies related to growing, replication and partnering outlined previously [20]. The potential for sustainability to diffuse throughout a community, or to other communities, or to nationally led initiatives should be acknowledged as a mechanism through which to deliver the socio-technical transition to a low-carbon society. Although, it may not be as immediately quantifiable as switching from petrol to electric vehicles, or from retrofitting aspects of the housing stock, the diffusion of sustainability as an idea that can influence the later development of community projects across a range of sectors has a significant role to play in the journey.

**Figure 1:**
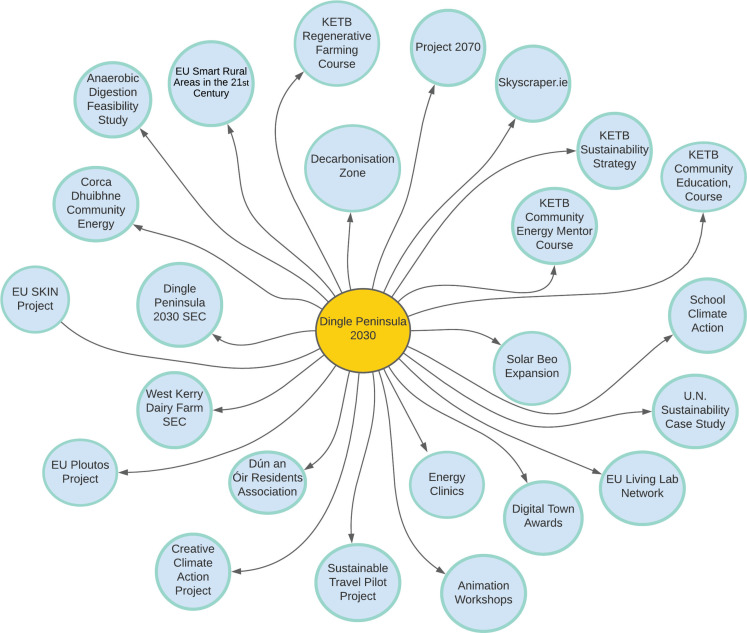
Visual representation of the diffusion of sustainability as part of *Dingle Peninsula 2030*.

Concerning *Dingle Peninsula 2030*, the range of different diffusions leads to a number of issues with relation to the theories, assumptions and constraints which would be needed within the model. For example, the factors influencing the diffusion of technology are vastly different from the diffusion of climate education. Alongside this, due to the increased national and international attention given to decarbonisation and sustainability, the external factors placed upon the diffusion process would also need to be attributed within the model. Further research, detached from the active nature of this research project’s relationship with *Dingle Peninsula 2030*, may prove insightful with relation to the diffusion of sustainability in the region. The need for a broader time frame for an in-depth investigation of diffusion must be noted here. With this in mind, the different aspects of the diffusion process have been highlighted in a visual contained in the results section (Fig. 1). We have outlined the key diffusion processes, giving an overview of each with information garnered from exploratory interviews, attendance of events and meetings and secondary desk research. These have then been assigned designations related to relevant SDGs.

## Results

### Outline of diffusions of sustainability from Dingle Peninsula 2030

Through this approach to highlighting emergent initiatives both within the community in question, and further afield, a range of diffusions of sustainability, influenced by *Dingle Peninsula 2030* have been outlined. In this section, these diffusions are highlighted through a visual aid and a table providing a contextual outline. The different initiatives have been linked to the SDGs. Due to the ongoing nature of the project, only a snapshot in time can be represented here with relation to the diffusion of sustainability. How the project and the emergent initiatives within it develop in the coming years will only become known with time. The information contained here is up to date as of January 2022.

Table 1 outlines the SDGs linked to diffusions to date. Two of the goals were not listed as they are crosscutting objectives of the overall project: SDG 11 (sustainable cities and communities) and 17 (partnership for the goals). The core focus of the initiative in energy (SDG 7) and climate (SDG 13) is clear, but there are importantly also social goals in education (SDG 4), decent work (SDG 8) and innovation (SDG 9).

**Table 1.** Diffusions of sustainability and how they align with the Sustainable Development Goals

Diffusion	Sector(s)	Linked SDG
1. Decarbonisation zone	Region chosen as county’s designated area for trialling climate mitigations measures	3, 7, 9, 12, 13
2. Corca Dhuibhne Community Energy	Group formed to work on energy related initiatives for community benefit on the Peninsula	7, 12, 13
3. *Dingle Peninsula 2030* SEC	Created as part of the national Sustainable Energy Community network with a focus on residential retrofits and energy production	7, 12, 13
4. West Kerry Dairy Farm SEC	A group of 100 farmers investigating solar production in the agricultural sector	6, 7, 8, 12, 13, 15
5. Dún an Óir Residents Association	55 members of residents’ association exploring collaborative energy efficiency upgrades	7, 13
6. Energy clinics	Facilitated through Dingle Hub, providing online advice on low-carbon technologies for home upgrades	7, 13
7. Solar Beo Expansion	Company established to provide clean energy upgrades	7, 8, 9, 13
8. KETB Community Energy Mentor Course	Course created providing training to 10 local people to act as energy mentors	4, 7, 13
9. KETB Regenerative Farming Course	Course developed on biological farming and regenerative practices	4, 13, 15
10. Anaerobic Digestion Feasibility Study	A group of stakeholders in the agricultural sector have formed a steering group to investigate the potential for anaerobic digestion in the area	7, 12, 13, 15
11. EU SKIN Project	An EU funded project investigating localised supply chains in the hospitality and agricultural sectors	3, 8, 15
12. EU Ploutos Project	An EU funded project investigating the use of sensors and data for environmental parameters in the agricultural sector	3, 8, 9, 15
13. EU Smart Rural Areas in the 21st Century	Selected as representative in Smart Rural 21 initiative, to promote and inspire villages/rural towns to develop and implement smart village approaches and strategies	3, 7
14. Creative Climate Action Project	Nationally funded project with local farmers to creatively look at ways in which farmers on the Peninsula can diversify to address climate change	12, 13, 15
15. Sustainable Travel Pilot Project	Steering group established to investigate numerous local transport initiatives	3, 9, 12, 13
16. Animation Workshop	Six-week course with secondary level students creating animations related to topics of sustainability	4
17. Digital Town Awards	Dingle awarded national digital town of the year	9
18. EU Living Lab Network	The Dingle Peninsula accepted into the EU Living Lab Network, highlighting local community approaches to sustainability and climate action	17
19. UN Sustainability Case Study	A UN case study created on *Dingle Peninsula 2030*	13, 17
20. School Climate Action	Working with secondary school students, ‘climate hacks’ were organised to devise decarbonisation pathways for transport, residential and energy production	4, 7, 13
21. KETB Community Education Course	Course developed through Kerry Enterprise and Training Board in relation to community education programmes	4, 13, 15
22. KETB Sustainability Strategy	Work on the Peninsula acted to inform the development of Kerry Education and Training Boards Sustainability strategy	13
23. Skyscaper.ie	Supported the emergence of a sustainable production company focused on high quality sustainable products such as clothing, office supplies and cutlery	9
24. Project 2070	Coastal adaptation project on the Maharees	13, (possibly 14 – coastal protection)

The adopted approach demonstrates the breadth of sustainability initiatives underway in the case study region, which could be considered an early sign of progress. However, the extent of the impact remains to be seen with how the various projects develop over time. Exactly how each contributes to the relevant SDGs would require closer monitoring of key indicators over much longer timescales.

## Discussion

### How can we understand the diffusion of sustainability in an ongoing community-based climate action initiative?

Following on from the outline of diffusions of sustainability related to *Dingle Peninsula 2030*, a discussion on the wider relevance of the investigation of diffusion takes place. This is related to the challenges and constraints of such an approach with relation to the wider significance of action research to the project, the role of *Dingle Peninsula 2030* as an intermediary for diffusion, and the potential for national and international diffusion of sustainability with a holistic approach taken to sustainability facilitated through the SDGs. Returning to *Dingle Peninsula 2030* as a frame through which to situate community as desire, the emergence of a wide range of different initiatives can be understood as the emergence of a wider desire for community.

### Diffusion of sustainability in action; challenges and constraints

The importance of community-level actions and initiatives for the delivery of climate action and sustainability more broadly has been acknowledged within policy [39,40], and is of central consideration within this series [41]. Through the initiation and establishment of a local sustainability project, the potential for diffusion throughout the community, to other communities and nationally led initiatives can be acknowledged as a mechanism through which to deliver the socio-technical transition to a low-carbon society. Investigating the diffusion of sustainability in this context can assist with this process of acknowledgement. This gives greater validity and support to community-led projects which can increase interactions between niche innovations and regime actors.

There are, however, several limitations that have been encountered that must be acknowledged. Firstly, the issue of attribution must be addressed. Aligning with the multi-level perspective [42], transitions operate across and between three levels: the niche, the regime and the landscape. Alongside this, understandings of the diffusion of innovations previously outlined can be incorporated, whereby diffusion can occur from internal, external, or mixed influence. Within the outline of the different initiatives contained within the results, influence has been attributed to *Dingle Peninsula 2030* (and within this the work of Dingle Hub, MaREI, NEWKD and ESB Networks). Despite this, the concept of mixed influence [24], must be acknowledged. External influence on the emergence of new initiatives has an important role to play. While the local project facilitates the spread of information, capacity building and trust development, the external influence of landscape narratives around sustainability, policy supports for initiatives and economic drivers on a global scale are also important parts of the mix. Absolute suggestions of attribution should be treated sceptically.

Another constraint is the relationship between the research approach taken and the established methods for investigating diffusion. When applying an engaged research methodology, whereby the research team is embedded within a ‘live’ project, seeking both practical problem-solving and the continued development of scientific knowledge, the ability to definitively outline the diffusion of sustainability, and within this the role of *Dingle Peninsula 2030*, is restricted. For example, the impact (e.g., emissions reductions) associated with key diffusions such as the community energy groups (2, 3 and 4 in Table 1) cannot be easily determined in the short-term as they have yet to bring a project to completion. Moreover, there is the issue of the embedded nature of the research, with researchers as active members of the project, contributions from the research approach and community actors become difficult to delineate.

The third key methodological challenge is determining the extent of the impact for each of the various initiatives. For energy and climate goals, the CO_2_ emission savings of technologies can be determined. This is possible for some of the technologies implemented to date, for example, tracking the number of solar photovoltaic (PV) installations, savings resulting from home energy improvements or electric vehicles [43]. However, as the majority of the projects listed in Table 1 are at a very early stage, it is not possible to determine the impact on key indicators at this time. Moreover, broader social benefits and capacity building, which are critical to the delivery of projects, cannot be so easily measured.

Through outlining the different diffusions, the foundation for further investigations which can build on what is presented here, analysing the diffusion of sustainability in a retrospective manner, has been set. Alongside the diffusions, more initiatives and projects will emerge, with the importance of mixed influence as outlined through the theoretical insights from studies of relevant diffusions of innovation. The landscape will change, *Dingle Peninsula 2030* will continue to develop or dissolve over time, and a range of initiatives will emerge and/or fade away. What we have set out here is an active understanding of the diffusion of sustainability in real time, which lays a foundation for continued study in this area both concerning the project in question and other similar initiatives nationally and internationally.

### Intermediaries for the diffusion of sustainability

The pitfalls of attribution and the need to incorporate theoretical understandings on mixed influence with regards to the diffusion of innovations are clear. Still, the importance of *Dingle Peninsula 2030* as it relates to the emergence of a range of sustainability initiatives both locally and nationally is self-evident. It was initiated as a collaborative project aiming to transition a geographical region in the Southwest of Ireland to a low-carbon, climate resilient, sustainable society by 2030. The project has sought to facilitate and support the emergence of different initiatives, and through this took an organic approach, allowing locally led initiatives to emerge rather than take a prescriptive approach.

Several different local efforts have emerged over the 3-year time frame of the project such as the anaerobic digestion project, the community energy mentor course and the West Kerry Dairy Farm Sustainable Energy Community (SEC) (see Table 1). The *Dingle Peninsula 2030* partnership, while supporting these different projects, also allowed for the emergence of local actors and organisations to involve themselves in different aspects of the initiatives. The diffusion of sustainability, as a core idea across the different projects, was influenced by *Dingle Peninsula 2030*, with sustainability central to its vision. From the initial partnership a large number of groups have formed around specific projects, with many of the diffusions outlined in Table 1 having independent steering groups or committees. An increasingly important issue is the question of governance and coordination between the various local groups. Within policy developments, importance is placed on supporting the emergence of vibrant communities that can lead the socio-technical transition within their regional context. Projects such as *Dingle Peninsula 2030*, whilst acting within their own agenda and vision, can operate as intermediaries between top-down policy and bottom-up initiative with relation to the diffusion of sustainability.

Despite the embedded nature within the local regional context, the organisational structure of *Dingle Peninsula 2030* which combines local groups (Dingle Hub and NEWKD) with a national body and research institute (ESB Networks and MaREI) builds the capacity to play a role between the top-down and bottom-up with relation to sustainable transitions. The diffusion of sustainability locally can be supported by the intermediary role played by regional collaborative projects such as *Dingle Peninsula 2030*. From a bottom-up perspective, intermediaries play a crucial role in stimulating and facilitating the emergence and continuity of grassroots initiatives [44]. Furthermore, they operate in a communicative role; ‘as traditional boundaries between actor groups are being eroded or redefined, intermediaries would appear to play an important role in communicating across cultures of compliance (state), of competition (market) and of collaboration (civil society)’ [45]. In relation to *Dingle Peninsula 2030*, the intermediate role is not the primary function, but the capacities of the collaborative grouping [16] enable it to be performed. This fits within the literature on intermediation whereby the intermediate role is played in balance with other roles and interests [46].

The overarching vision of *Dingle Peninsula 2030*, driven by the desire for a more environmentally and economically sustainable future on the Dingle Peninsula, means intermediate functions which facilitate initiatives aligned with this vision are mutually beneficial. By way of example, Dingle Hub’s support of the West Kerry Dairy Farm SEC in developing their application to Sustainable Energy Authority of Ireland or MaREI’s support with relation to the initiation of the anaerobic digester feasibility study represent two amongst many when it comes to the provision of intermediary supports for emergent initiatives. The diffusion of sustainability is predicated on this intermediary role.

### Diffusion beyond the regional context

The national diffusion of sustainability has been referenced within the results with relation to the education initiatives and their emergent influence within the national system. Alongside this, there have been a number of outreach activities and events which seek to disseminate the story of *Dingle Peninsula 2030* both nationally and internationally [19]. Within an engaged research approach, the drive for societally relevant and impactful research interventions must not be restricted to the local context. With this in mind, MaREI has played a key role in the dissemination of findings and also in relation to network building events beyond the regional context. Dingle Hub and ESB Networks have also led several events and meetings which align with these goals. Some of these are outlined as they relate to the diffusion of sustainability beyond the regional context.

The project has received extensive national exposure, including:

- Being one of six case studies featured in the National Broadcaster’s *Change Makers in Irish Universities* television series.
- A central case study in the Irish University Association Campus Engage ‘*Engaged Research: Train the Trainer*’ course.
- An exemplar engaged research project in the Royal Irish Academy Workshop and White Paper entitled ‘*Better together: knowledge co-production for a sustainable society*’
- Knowledge sharing with the Sustainable Communities Training Programme (SECAD) (3 × training sessions between September and December 2021).
- Presenting at Ireland’s first energy planning conference organised by Codema in November 2021.
- Launch by the Minister for Community, Joe O’Brien, of the Demographic and Socio-Economic Profile of the Dingle Peninsula.
- Recognised as a leading case study for transdisciplinary, action-orientated research in Science Foundation Ireland’s annual report.

It has also made significant contributions to the international science–policy interface:

- Participation in events at the UNFCCC COP26 Climate Conference, Glasgow 2021 including travelling in an electric vehicle to Glasgow for COP26 and documenting the journey via a blog, social media posts and a video.
- Being showcased at the EPRI European Workshop Week 2021 on Advancing Energy Communities.
- A dialogue session was held at International Sustainable Transitions (IST) conference 2020. The title of the session was ‘*Facilitating sustainable transitions through innovative governance*’, and convened a number of panellists to share experiences from different viewpoints. These included the manager of the Dingle Hub (Dingle Hub), an expert in social innovations and a representative from the UN.
- Hosting an armchair chat with members of the *Dingle Peninsula 2030* group to discuss their experiences with the project to date. The event was held in November 2020 and was attended by over 200 people, many from within the national SEC Network.
- The *Dingle Peninsula 2030* project was outlined by the UN Regional Information Centre for Western Europe (UNRIC) as an exemplar project on their website.

A significant contribution to knowledge sharing is the innovative ‘learning brief’ template developed as a way to collaboratively document reflections and learnings. These emerged as a useful means to capture important learnings on different projects or elements of the initiative in order to inform policy and community action elsewhere. They cover a variety of topics such as the ‘Climate Hacks’ with schools [47], value of collaboration [48] and trialling of electric vehicles [49].

## Conclusion

This paper has demonstrated the wide variety of sustainability initiatives that can emerge from community-based collaborations, through outlining a case study. The *Dingle Peninsula 2030* partnership began with a very clear focus on the electrification of heating and transport, which is a central part of climate mitigation policy in Ireland. However, as illustrated through the mapping of emerging local initiatives to the SDGs, this has diffused into a rich diversity of sectors. The multi-stakeholder collaboration has acted as a springboard for local efforts, which stands to highlight the important role of intermediaries to bridge top-down and bottom-up objectives. The initial assessment of the diffusion of sustainability can provide a useful starting point for understanding the impact of such projects. Several challenges associated with this assessment of an emergent and evolving project have also been outlined. Finally, the ways in which the project has attempted to diffuse beyond the case study region demonstrates the valuable contribution of such efforts to policy and action elsewhere. Again, here the importance of intermediaries, and in particular researchers, is highlighted. The holistic approach to sustainability as outlined through the SDGs has been well represented through the *Dingle Peninsula 2030* project. The diffusion of sustainability can act as a concept which enables collaborators to track emergent initiatives across a wide scope of sectors as outlined by the SDGs. Community led climate-action projects have potential to meet a wider spectrum of sustainability topics due to the diversity of interests and values contained within community contexts.

## Data Availability

The datasets generated during and/or analysed during the current study are available from the corresponding author on reasonable request.
